# Cooling Under a Blanketrol System Versus Cooling With an Arctic Sun Thermoregulation System (CATS) for Neonates Undergoing Therapeutic Hypothermia

**DOI:** 10.7759/cureus.59634

**Published:** 2024-05-04

**Authors:** Mahmoud A Ali, Muppala Prasanth Raju, Tara Lyngaas, Venkata Raju, Shreya Jatla, Nguyen Nguyen, Niraj Vora, Madhava Beeram, Vinayak Govande

**Affiliations:** 1 Neonatology, West Virginia University, Morgantown, USA; 2 Pediatrics, Baylor Scott & White Health, Temple, USA; 3 Neonatal Intensive Care Unit (NICU), Baylor Scott & White Health, Temple, USA; 4 Neonatology, Baylor Scott & White Health, Temple, USA; 5 Pediatrics, University of Texas at Austin, Temple, USA

**Keywords:** nicu, thrombocytopenia, thermoregulation, temperature, arctic sun, blanketrol, cooling, hypoxic ischemic encephalopathy, neonatal encephalopathy, therapeutic hypothermia

## Abstract

Background

Despite evidence suggesting improved outcomes in neonates with hypoxic-ischemic encephalopathy (HIE) treated with therapeutic hypothermia (TH), data on the impact of temperature variability during cooling and its association with clinical outcomes remain limited.

Objective

To compare the efficacy and ease of use of two different cooling systems, the Arctic Sun (Medivance, Inc., Louisville, CO) vs. the Blanketrol III (Gentherm Medical, Cincinnati, OH) on achieving TH, temperature variability, and clinical outcomes in neonates with HIE undergoing TH.

Methods

This study was conducted at the Baylor Scott and White Medical Center's Level IV NICU. The study employed a retrospective cohort design, comparing infants treated with the Arctic Sun device (from December 2020 to August 2021) to a historical cohort treated with the Blanketrol system (from January 2017 to November 2020). Both groups were evaluated for clinical characteristics, patients’ outcomes, and ease of use of the cooling devices. Ease of use was assessed through a self-developed survey administered to NICU nurses. Core body temperatures throughout the cooling course were documented at four-hour intervals, including induction, maintenance, and rewarming phases.

Results

Twenty-two infants were cooled using the Arctic Sun system, and 44 infants were cooled with the Blanketrol device. Median birth weight and gestational age were comparable. There were no significant differences in one-minute and five-minute appearance, pulse, grimace, activity, and respiration (APGAR) scores. The Arctic Sun group had a significantly higher rate of maternal morbidities, including diabetes and placental abruption. Although the median temperature achieved with both devices was 33.5°C, temperature variability was significantly greater with the Blanketrol device (p = 0.03). Thrombocytopenia rates were statistically different between the groups (9% in Arctic Sun vs. 38% in Blanketrol, p = 0.001). Although the Blanketrol group had higher rates of disseminated intravascular coagulation (48% vs. 37%), hypercalcemia (23% vs. 5%), and subcutaneous fat necrosis (7% vs. 5%), these differences were not statistically significant. A nurses' survey on ease of use revealed a strong preference for the Arctic Sun cooling system. Over 85% of nurses found it easier to learn and set up and required less manual intervention than the Blanketrol device.

Conclusions

Gel adhesive pad-based TH is a potentially superior modality to traditional water-circulating cooling devices. These pads offer advantages in user-friendliness, improved temperature control precision, and potentially reduced adverse event profiles.

## Introduction

Hypoxic-ischemic encephalopathy (HIE) is the most common cause of neurological disability and mortality in newborns (>1 million neonatal deaths globally each year) [1], with a prevalence of approximately 1-4 per 1,000 neonates in developed countries and ~26 per 1,000 neonates in developing countries [1-3]. About 35% of neonates with neonatal encephalopathy (NE) develop long-term neurodevelopmental sequelae, such as mental retardation, epilepsy, cerebral palsy, and learning disabilities, while 25% die within the first two years of life [2-4]. Therapeutic hypothermia (TH) is the only and current standard of treatment for moderate or severe cases of HIE in term and near-term infants born 36 weeks or greater [3,4].

Although TH does not eliminate mortality and disability, it demonstrably reduces their risk in patients with moderate-to-severe HIE [5]. Despite resource constraints limiting its availability in some hospitals, TH treatment is used worldwide; several hospitals utilize simple external cooling devices such as cooling blankets and ice packs [1,2].

While simple, these cooling methods were not designed to cool critically ill patients rapidly and do not give precise body temperature control during hypothermia induction, maintenance, and reversal [1,2,5]. Most devices used for cooling were initially developed for and used in postcardiac surgery in adult patients [6]. These devices are used for both the inductive and maintenance phases of hypothermia. However, performance metrics were seldom compared between devices. Some studies have attempted to compare devices but not at the same center [7].

External water-circulating cooling blankets (Blanketrol III; Gentherm Medical, Cincinnati, OH) are commonly used in the NICU [1,3,7]. Gel-coated adhesive cooling pads, which the Arctic Sun (Medivance, Inc, Louisville, CO) device uses, are relatively new in this cohort [4]. The Arctic Sun system is a temperature management system approved by the US Food and Drug Administration. It differs from traditional cooling blankets in three ways: 1) it produces higher cold fluid flow rates, 2) it uses conductive, adhering gel pads for cooling-whereas Blanketrol III employs a special water-circulating blanket to regulate temperature-and 3) it has a precise temperature feedback-control system that may enable faster-cooling induction and better temperature control during hypothermia maintenance and rewarming [8,9].

This study compared the Arctic Sun blanket to traditional cooling blankets (Blanketrol III) for TH induction and maintenance following HIE. We hypothesize that the Arctic Sun device is more effective at temperature regulation and user-friendly than the Blanketrol III.

## Materials and methods

Methods

This historical cohort study was conducted at the Level IV NICU of Baylor Scott & White Health Medical Center, Temple, Texas. Before the study, the institution had a protocol for cooling infants diagnosed with HIE. The institutional review board approved the protocol. The study included 44 infants cooled with Blanketrol III and 22 with the Arctic sun system. Inclusion criteria comprised any infant diagnosed with HIE and requiring TH. We used the electronic medical record (EMR) system (Epic) to collect information on the data variables. The Blanketrol III used a rectal probe to measure core temperature, whereas the Arctic sun used an esophageal probe. Infants cooled with Blanketrol III were identified by chart review. We included infants on whom TH using Blanketrol III was performed from January 2017 to November 2020. Three arctic sun units were operationalized in December 2020, and this system cooled infants until August 2021, when the study period ended.

Primary outcomes were temperature variability assessment, TH complication, and machine operating ease.

The clinical team electronically downloaded the Arctic Sun system data, and data were captured every minute for the whole cooling period. The clinical team captured the Blanketrol III system data from the entered data into the EMR at several time intervals (every 15 minutes, every 30 minutes, or sometimes an hour too), depending on the need to assess for extreme variability and different phases. To standardize the comparison, the clinical team captured a four-hour temperature and then took the average temperature. Once the mean was known, the median was taken to assess variability throughout the TH period. Thrombocytopenia was defined as a platelet count below 150,000 after cooling began, and there was no baseline thrombocytopenia before cooling. Disseminated intravascular coagulation (DIC) was defined as the fibrin degradation products present in addition to prolonged PT. Hypercalcemia was defined as serum levels >11 mg/dl. Subcutaneous fat necrosis (SCFN) was assessed clinically.

We also sent out a survey to assess the NICU nurses' opinions of the two devices. The survey, created on REDCap, had five questions on the machines' ease of use, and it was distributed internally to all the nurses who had worked in the NICU on both devices over the study period (Appendix 1).

Statistical analysis

Differences in the categorical variables were assessed using the chi-square test, and those with the continuous variables were evaluated using the Wilcoxon rank sum test. Descriptive data are presented as mean. Data are presented as the number of patients (n) and percentage. The nurses' survey is presented as a percentage. P < 0.05 was considered statistically significant.

## Results

We prospectively enrolled 23 infants admitted with HIE for TH. One patient was excluded because of withdrawal from the study. Complete data were available for 22 infants cooled using the Arctic Sun device. From the chart review, data on 44 cooled infants using Blanketrol III were included. Overall, 66 patients were available for comparison, 22 from the Arctic Sun and 44 from the Blanketrol III. Both the groups were similar except for statistically significant differences in the maternal gestational diabetes rates (6/27, 1/2, p = 0.002) (Table 1).

**Table 1:** Baseline characteristics of the studied population GBS: Group B Streptococci; APGAR: appearance, pulse, grimace, activity, and respiration

Subject demographics	Arctic Sun (n=22)	Blanketrol III (n=44)	P value
Male	9 (41)	22 (50)	0.485
Birth weight, kg	3.04 (2.37-5.75)	3.24 (2.07-4.75)	0.59
Gestational age, weeks	38.3 (36.1-40.4)	39.1 (36.0-41.2)	0.11
APGAR 1 min	1 (0-3)	2 (0-6)	0.22
APGAR 5 min	4 (0-6)	4 (0-8)	0.18
Race			0.527
Caucasian	15 (68)	27(61)	
African American	6 (27)	11 (25)	
Other	1 (5)	6 (14)	
Inborn	8 (38)	17 (39)	0.967
Survival rate	22 (100)	42 (95)	0.31
C-section	15 (68)	28(64)	0.715
Maternal characteristics:			
Abruptio placenta	2 (9)	0	0.1
Infant of diabetic mother	6 (27)	1 (2)	0.002
Preeclampsia	5 (23)	6 (14)	0.35
Maternal GBS	5 (23)	9 (20)	0.831
Chorioamnionitis	2 (9)	6 (14)	0.594

The common complications associated with TH are presented in Table 2. Thrombocytopenia was significantly higher in the Blanketrol III group (38% vs. 9%). The temperature variability, too, was included as a common adverse event and was noted to have greater variation in the Blanketrol III cohort (p = 0.03). The incidence of subcutaneous fat necrosis (SCFN), hypercalcemia, and DIC were all higher in the Blanketrol III group. However, the differences were statistically insignificant (p value was 0.73, 0.06, and 0.38, respectively) (Table 2).

**Table 2:** Common complications associated with TH DIC: disseminated intravascular coagulopathy *Throughout cooling at a four-hour interval—starting at four hours after cooling initiation—this also included rewarming and maintenance phases

Temperature variability and complications	Arctic Sun (n=22)	Blanketrol III (n=44)	P value
Temperature*	33.50 (33-36.5)	33.5 (31.7-35.5)	0.03
Subcutaneous fat necrosis	1 (5)	3 (7)	0.72
Hypercalcemia	1 (5)	10 (23)	0.06
DIC	8 (37)	21 (48)	0.381
Thrombocytopenia	2 (9)	23 (38)	0.001

The nurses significantly favored the Arctic Sun thermoregulation system on all the five parameters assessed (i.e., learnability, setup simplicity, post-setup intervention frequency, complexity/troubleshooting, and overall usability) (Table 3).

**Table 3:** The survey results sent out to the NICU nurses

Nurses survey based on ease of use between the cooling machines. Total Respondents, N=41	Arctic Sun is preferred or greatly preferred (%)	Blanketrol III is preferred or greatly preferred (%)
Considering how difficult it was to learn how to use the device or how often I needed to ask for help	85.4	4.9
When I think about how much time it takes to properly set up the device and initiate therapy	87.8	7.3
Recalling how often I was required to manually alter device settings during my shift	90.3	4.9
Based on the quantity and/or complexity of troubleshooting attempts during a therapy session	78.1	9.8
Overall, weighing all "ease of use" characteristics	90.2	4.8

## Discussion

Both cooling devices utilized in this study, Blanketrol III and Arctic Sun, effectively provided targeted TH. The median temperature obtained via both devices over the 72 hours of cooling, including the induction, maintenance, and rewarming phase, was 33.5°C. The data were along the lines of existing literature, and studies were done in postcardiac infants and neonates undergoing TH with other devices [10]. The temperature maintenance was well-regulated with minimal variation with the Arctic sun system, and it had fewer associated complications. The nurses favored the Arctic sun for its ease and involved less hands-on manipulation.

Our study adds to earlier studies by Heard et al. who showed that gel-cooling pads were more effective in temperature control in a randomized control study of 64 patients. The target temperature was reached faster, or within four hours (p = 0.01) [9]. Mayer et al. studied hyperthermia in patients on Blanketrol III and Arctic Sun in 47 patients. The Arctic sun group had a significantly lower fever burden (p < 0.001) [11]. Notably, some of the research covered temperature variability and found trends toward better results linked to better temperature control [9,11,12]. Tomte et al. reported that invasive vs. noninvasive cooling resulted in survival rates of 45% vs. 38% with good neurologic function (p = 0.345) [13]. Oh et al. compared the outcomes between 559 patients with surface cooling and 244 individuals who received endovascular cooling (Blanketrol, Meditherm, or Arctic Sun) [14]. Positive neurologic results were 35.4% vs. 25.6% (p = 0.01). After propensity score matching in 360 patients, the numbers were 35% vs. 30%, indicating that the groups were not adequately matched (p = 0.31) [14]. Even though none of the changes in the aforementioned studies approached statistical significance when considered collectively, this suggests that more efficient cooling (i.e., less temperature fluctuation) could confer more substantial protective effects [12-15]; future meta-analyses may help to confirm or deny this possibility.

Nielsen et al. emphasized the potential significance of precise temperature control [16]. Their investigation suggests that maintaining accurate temperature management might become even more critical if institutions adopt alternative target temperature ranges for TH from 33.0°C to 36.0°C. As a result, keeping a constant temperature is perhaps more crucial and challenging at 36.0 °C than at 33.0 °C. This was also demonstrated by another study from Australia, after changing the target temperature from 33.0°C to 36.0°C, patients spent less time at the target temperature (87% vs. 50%, p 0.001), had higher rates of fever (0% vs. 19%, p = 0.03), and had trends toward worse outcome (survival 71% vs. 58%, p = 0.31) [17]. Compared to a target temperature of 32-33°C, a 36°C target temperature may be more challenging to maintain and may call for more potent and precise equipment [16,17].

Perinatal asphyxia can result in reductions in levels of some factors of the extrinsic pathway, which has been associated with thrombocytopenia and DIC [18]. Our analysis revealed no significant difference in the incidence of DIC between the study groups. However, a novel finding of this study is the significant difference in the incidence of thrombocytopenia between the study groups employing different cooling devices. A comprehensive literature search identified no prior reports on this specific association. Our findings warrant further investigation into the potential association between specific cooling devices used in TH and the severity and complications of thrombocytopenia in neonates with HIE. Future studies with larger sample sizes are needed to confirm these observations.

The current study did not find significant differences in the incidence of subcutaneous fat necrosis or hypercalcemia between the study groups. These findings align with prior literature, suggesting that SCFN remains a potential adverse event in neonates with HIE undergoing TH [19,20].

A pattern of preference emerged among participating nurses in the current study for the Arctic Sun thermoregulatory system during TH in neonates. This favorability might be attributed to the device's user-friendly design, potentially leading to less frequent operator intervention.

The current study design minimizes the potential for a Hawthorne effect as the primary outcomes (temperature variability, hypercalcemia, DIC, and SCFN) are objective and independent of the clinical team's awareness, and while the survey on the Arctic Sun device's ease of use might be susceptible, its influence on overall findings is likely limited. Our study is limited in that it does not report the cohort's neurodevelopmental outcomes because of scarce resources. However, our study is the first of a kind comparing two cooling modalities, in neonates, in the same center. A single-center experience avoids variations in several areas: cooling system type, individual NICU practices, cooling protocol, transport protocol (if necessary), and laboratory investigation protocols.

## Conclusions

Gel adhesive pad-based (Arctic Sun) TH is a potentially superior modality to traditional water-circulating cooling devices (Blanketrol III). These pads offer advantages in user-friendliness and improve temperature control precision. Further investigation through larger-scale studies is warranted to definitively assess both short-term and long-term clinical outcomes associated with gel pad technology.

## Appendices

**Table 4:** Appendix 1: Cooling blankets survey

Please complete the survey below. Thank you.
1) Considering how difficult it was to learn how to use the device or how often I needed to ask for help.
Blanketrol is greatly preferred.
Blanketrol is preferred.
Blanketrol is slightly preferred.
I have no preference.
Artic Sun is slightly preferred.
Artic Sun is preferred.
2) When I think about how much time it takes to properly set-up the device and initiate therapy.
Blanketrol is greatly preferred.
Blanketrol is preferred.
Blanketrol is slightly preferred.
I have no preference.
Artic Sun is slightly preferred.
Artic Sun is preferred.
Artic Sun is greatly preferred.
3) Recalling how often I was required to manually alter device settings during my shift.
Blanketrol is greatly preferred.
Blanketrol is preferred.
Blanketrol is slightly preferred.
I have no preference.
Artic Sun is slightly preferred.
Artic Sun is preferred.
Artic Sun is greatly preferred.
4) Based on the quantity and/or complexity of troubleshooting attempts during a therapy session.
Blanketrol is greatly preferred.
Blanketrol is preferred.
Blanketrol is slightly preferred.
I have no preference.
Artic Sun is slightly preferred.
Artic Sun is preferred.
Artic Sun is greatly preferred.
5) Overall, weighing all "ease of use" characteristics.
Blanketrol is greatly preferred.
Blanketrol is preferred.
Blanketrol is slightly preferred.
I have no preference.
Artic Sun is slightly preferred.
Artic Sun is preferred.
Artic Sun is greatly preferred.

## References

[1] Chiang MC, Jong YJ, Lin CH (2017). Therapeutic hypothermia for neonates with hypoxic ischemic encephalopathy. Pediatr Neonatol.

[2] Akula VP, Joe P, Thusu K (2015). A randomized clinical trial of therapeutic hypothermia mode during transport for neonatal encephalopathy. J Pediatr.

[3] Ranjan AK, Gulati A (2023). Advances in therapies to treat neonatal hypoxic-ischemic encephalopathy. J Clin Med.

[4] Broessner G, Fischer M, Schubert G, Metzler B, Schmutzhard E (2012). Abstracts of the 2nd Innsbruck Hypothermia Symposium. Crit Care.

[5] Dallera G, Skopec M, Battersby C, Barlow J, Harris M (2022). Review of a frugal cooling mattress to induce therapeutic hypothermia for treatment of hypoxic-ischaemic encephalopathy in the UK NHS. Global Health.

[6] Bernard SA, Gray TW, Buist MD, Jones BM, Silvester W, Gutteridge G, Smith K (2002). Treatment of comatose survivors of out-of-hospital cardiac arrest with induced hypothermia. N Engl J Med.

[7] Sonder P, Janssens GN, Beishuizen A (2018). Efficacy of different cooling technologies for therapeutic temperature management: a prospective intervention study. Resuscitation.

[8] Warttig S, Alderson P, Campbell G, Smith AF (2014). Interventions for treating inadvertent postoperative hypothermia. Cochrane Database Syst Rev.

[9] Heard KJ, Peberdy MA, Sayre MR (2010). A randomized controlled trial comparing the Arctic Sun to standard cooling for induction of hypothermia after cardiac arrest. Resuscitation.

[10] Coggins SA, Haggerty M, Herrick HM (2021). Post-cardiac arrest physiology and management in the neonatal intensive care unit. Resuscitation.

[11] Mayer SA, Kowalski RG, Presciutti M (2004). Clinical trial of a novel surface cooling system for fever control in neurocritical care patients. Crit Care Med.

[12] Deye N, Cariou A, Girardie P (2015). Endovascular versus external targeted temperature management for patients with out-of-hospital cardiac arrest: a randomized, controlled study. Circulation.

[13] Tømte Ø, Drægni T, Mangschau A, Jacobsen D, Auestad B, Sunde K (2011). A comparison of intravascular and surface cooling techniques in comatose cardiac arrest survivors. Crit Care Med.

[14] Oh SH, Oh JS, Kim YM (2015). An observational study of surface versus endovascular cooling techniques in cardiac arrest patients: a propensity-matched analysis. Crit Care.

[15] Finley Caulfield A, Rachabattula S, Eyngorn I (2011). A comparison of cooling techniques to treat cardiac arrest patients with hypothermia. Stroke Res Treat.

[16] Nielsen N, Wetterslev J, Cronberg T (2013). Targeted temperature management at 33°C versus 36°C after cardiac arrest. N Engl J Med.

[17] Bray JE, Stub D, Bloom JE (2017). Changing target temperature from 33°C to 36°C in the ICU management of out-of-hospital cardiac arrest: a before and after study. Resuscitation.

[18] Oncel MY, Erdeve O, Calisici E, Oguz SS, Canpolat FE, Uras N, Dilmen U (2013). The effect of whole-body cooling on hematological and coagulation parameters in asphyxic newborns. Pediatr Hematol Oncol.

[19] Grass B, Weibel L, Hagmann C, Brotschi B (2015). Subcutaneous fat necrosis in neonates with hypoxic ischaemic encephalopathy registered in the Swiss National Asphyxia and Cooling Register. BMC Pediatr.

[20] Zifman E, Mouler M, Eliakim A, Nemet D, Pomeranz A (2010). Subcutaneous fat necrosis and hypercalcemia following therapeutic hypothermia--a patient report and review of the literature. J Pediatr Endocrinol Metab.

